# Extramural Interments.—Practical Application of Charcoal to Burial Purposes

**Published:** 1855-06

**Authors:** 


### JOURNAL OF PUBLIC HEALTH.

JUNE 1855.

### EXTRAMURAL INTERMENTS.—PRACTICAL APPLICATION OF CHARCOAL TO BURIAL PURPOSES.

The question relating to extramural interments, recently brought before the House of Lords by the Lord Bishop of London, is one which we had long foreseen would of necessity require to be carefully considered by the legislator. Few questions, indeed, are at this moment of greater importance to the poor. The New Metropolitan Burial Act, while having for its objects the health and well being of the people, has, in its practical working, met with various serious difficulties, which possibly were not foreseen at the time when it came into force. The leading difficulty implied is, that the poor, amongst whom the mortality is greatest, find themselves utterly unable to meet the expenses of funerals for their deceased friends, whenever a considerable sum of money is required for conveying the dead to extramural burial places, far removed from the homes of those who have sustained the bereavements.

This unfortunate difficulty has now arrived at a crisis, which renders further silence impossible; and the community generally, but especially the poor, owe deep thanks to the Right Reverend Prelate for having so ably brought the subject before the House of Peers. The remarks made by the Bishop, by Lord Shaftesbury, and by other speakers, as to the demoralising and sanitary evils caused by the Act in the eastern districts of London, we ourselves can fully verify from personal observation and experience. We have known a deceased person kept for at least ten days in a single room, itself the absolute home of a family,—simply that time might be allowed for a sufficient sum of money to be collected to enable the relatives to meet the expenses incident to an interment, which expenses have proved a serious injury to the poor for many weeks afterwards; indeed, it sometimes happens in these cases, that loans, which of all things most burden the poor man, have to be incurred, to be repaid afterwards at great sacrifices. For these and other reasons, we believe that extramural interments can never be so carried out, as to meet the necessities of the lower classes.

How the dead shall be disposed of remains, therefore, still a great problem: one which in all ages of the world, and amongst all classes of mankind, has been ever considered unsolved.

It appears to us plainly, that the point to be aimed at is not to remove the bodies of deceased persons far away into rural districts, and deposit them there to undergo slow dissolution, near places which in future times may be covered by human habitations, but rather to invent some simple process, which, without doing away with proper burial grounds accessible to towns, and without doing violence to the better feelings of the community, shall permit of a form of burial, in which the decay of the animal structures shall go on rapidly without giving rise to dangerous exhalations. We are further of opinion, that the experimental researches on the decomposing properties of charcoal, lately so ably and satisfactorily originated and carried out by Dr. Stenhouse, indicate clearly a means for accomplishing an end so desirable. It is now a fully established fact, that a portion of dead animal substance, if surrounded by a thin layer of wood charcoal in powder, is quickly transformed into various elementary states, which, through the medium of the charcoal, enter as quickly into new combinations, perfectly innocuous in character; and there seems to be no reason why so simple a process might not be at once adopted on a grand scale for burial purposes.

If it were absolutely required, there would indeed be no mechanical reason why properly constructed burial grounds of charcoal itself might not be formed. But this plan, which would necessarily involve a great outlay at first, is, we take it, quite unnecessary. The only process that is required would be to surround the bodies of the dead, before they were finally enclosed, with a layer, some four inches thick, of finely powdered wood charcoal. As under a mistaken chemical notion, lime is very commonly and uselessly employed in a similar way, there is nothing startling or objectionable in this suggestion; nor would the poor, as we believe from an intimate knowledge of their feelings, raise any objections to its application, if they were taught that it had to do with the health of the community.

To carry out the details of this process effectually, however, a legislative enactment would be required, rendering it compulsory, and leaving it to some public officer to enforce the law.

An objection may, perhaps, be raised that the proceeding here referred to would also involve expense; and this is no doubt the case. But if so large a project were set on foot, the manufacture and importation of charcoal would become an important commercial concern, and its cost would be reduced to a minimum as regards the expenditure involved in one interment. Plans might be set on foot in fact for supplying the poor with it gratuitously from the union workhouse, or some other depôt; while the rich might much better expend a few shillings for so beneficial a purpose, than on those useless trappings and housings, by display of which they too often convert a solemn ceremony into an absurd pageant. Of course, in making this suggestion, we are in no way advocating the re-opening of crowded burial grounds; but we would recommend the formation of new burial grounds, accessible to the poor, and *constructed on scientific principles*, a point that has never yet been practically insisted on even in extramural cemeteries.

There is one other point connected with the Bishop of London's speech to which, as scientific writers, we should not refer, did it not bear on the sanitary question. His Lordship deplores that the New Metropolitan Act lessens seriously the incomes of the working clergy by stopping the influx of burial fees. He states that, in the parish of Whitechapel, the income of the incumbent has from this cause alone fallen from £600 to £300 a year. For our parts, we regret that any clergyman should receive so large a sum as £300 annually for burials, much of which must of necessity come from sources where its loss is most felt. We think it would be more dignified and useful if the parochial clergy were remunerated for their services in relation to burials from some general fund; and we know positively that the burial fee, small though it may be, cannot always be got together by the poor man at a short notice. In a sanitary sense, therefore, this, like extramural interment, has its serious evils amongst the poor.

The question relating to the proper construction of burial places involves many mechanical and chemical details, into which we cannot enter on the present occasion.

